# Phytochemical profiling and in vitro screening for anticholinesterase, antioxidant, antiglucosidase and neuroprotective effect of three traditional medicinal plants for Alzheimer’s Disease and Diabetes Mellitus dual therapy

**DOI:** 10.1186/s12906-018-2140-x

**Published:** 2018-03-02

**Authors:** Mohan Penumala, Raveendra Babu Zinka, Jeelan Basha Shaik, Suresh Kumar Reddy Mallepalli, Ramakrishna Vadde, Damu Gangaiah Amooru

**Affiliations:** 1 Department of Chemistry, Yogi Vemana University, Kadapa, Andhra Pradesh India; 2 Department of Biotechnology and Bioinformatics, Yogi Vemana University, Kadapa, Andhra Pradesh India

**Keywords:** *Buchanania axillaris*, *Hemidesmus indicus*, *Rhus mysorensis*, Phytochemical profile, Antioxidant activity, Anticholinesterase activity, Antidiabetic activity

## Abstract

**Background:**

Extensive epidemiological and clinical studies revealed that Alzheimer’s Disease (AD) and Type 2 Diabetes Mellitus (T2D) are most likely to appear simultaneously in aged people as T2D is a major risk factor for AD. Therefore, development of potential multifunctional agents for dual therapy of AD and T2D has received much attention. *Buchanania axillaris*, *Hemidesmus indicus* and *Rhus mysorensis* have been used extensively in popular medicine. The present study was aimed at phytochemical profiling and evaluating multifunctional ability of titled plants in the AD and T2D dual therapy.

**Methods:**

Methanolic extracts and their derived fractions were evaluated for their inhibitory capacities against acetylcholinesterase (AChE) & butyrylcholinesterase (BuChE), and α– & β–glucosidase besides kinetic analysis of inhibition using methods of Elmann and Shibano, respectively. Antioxidant potency of active fractions was assessed by their DPPH and ABTS radical scavenging activities. Active fractions were tested by the MTT assay to verify cytotoxicity and neuroprotective ability in human nueroblastoma cell lines. Phytochemical screening was done with the aid of spectrophotometric methods.

**Results:**

All the methanolic extracts of test plants (BAM, HIM, RMM) showed concentration dependent inhibitory activities against AChE, BuChE, α– and β–glucosidase enzymes. Subsequent fractionation and evaluation revealed that chloroform fractions BAC, HIC and RMC with IC_50_ values of 12.29±2.14, 9.94±2.14, 16.65±1.99 and 27.38±1.24; 28.14±0.9, 5.16±0.22, 11.03±0.5 and 87.64±15.41; 41.35±1.6, 15.86±7.3, 26.04±0.37 and 25.33±0.3 were most prominent with regard to inhibition potential against AChE, BuChE, α– and β–glucosidase, respectively. Kinetic analysis of these active fractions proved that they disclosed mixed-type inhibition against AChE, BuChE, α– and β–glucosidase enzymes. In the MTT assay, active fractions BAC, HIC, RMC showed significant cell viability at high concentrations (400 μg). Moreover, in MTT assay, the active fractions displayed excellent neuroprotective effects against oxidative stress induced cell death and significant cell viability in SK N SH cells at all concentrations.

**Conclusion:**

The strong anticholinesterase, antiglucosidase, antioxidant and neuroprotective activities of methanolic extracts and their derived chloroform fractions indicate the potential of *Buchanania axillaris*, *Hemidesmus indicus* and *Rhus mysorensis* as multifunctional therapeutic remedies for the dual therapy of T2D and AD.

**Electronic supplementary material:**

The online version of this article (10.1186/s12906-018-2140-x) contains supplementary material, which is available to authorized users.

## Background

Alzheimer’s disease (AD) is a chronic neurodegenerative irreversible brain disorder that affects daily living through memory loss and cognitive impairment [1, 2]. AD is affecting 46.8 million people throughout the world and this number is likely to get doubled by 2030 due to lack of effective treatment [3]. Although the etiology of AD remains elusive, multiple factors, such as low levels of acetylcholine (ACh), oxidative stress, accumulation of misfolded amyloid-β (Aβ) and dyshomeostasis of biometals have been considered to play definitive roles in the pathophysiology of AD, and several hypotheses based on these factors have been proposed to explain the mechanism of AD pathogenesis [4, 5]. According to the “cholinergic hypothesis”, acetylcholinesterase (AChE) acts primarily as a regulatory enzyme at cholinergic synapses, while butyrylcholinesterase (BuChE) an enzyme closely related to AChE serves as a co-regulator of cholinergic neurotransmission by hydrolysing ACh. AChE and BuChE dual inhibition have been documented as critical targets for the effective management of AD by an increase the availability of ACh in the brain regions [6–8].

Type 2 Diabetes Mellitus (T2D) is an age-related metabolic disorder with complex etiology and affecting 10% population across the world [9]. According to World Health Organization, the situation is most vulnerable in India, China and the United States with large number of affected individuals [10]. T2D is characterized by cellular insulin resistance, chronic inflammation and several metabolic abnormalities [11]. T2D causes abnormal absorption of glucose into the blood that creates a condition called hyperglycemia and results in serious complications in various organ systems like liver, heart, kidney, retina and brain [12, 13]. One of the therapeutic approaches to decrease postprandial hyperglycemia is the inhibition of carbohydrate hydrolyzing enzymes, α– and β–glucosidases (α– and β–Glu), thereby delaying glucose digestion in the digestive tract [14].

Conversely, epidemiological and clinical studies have revealed the possible pathophysiological links between T2D and AD. According to reports, T2D is considered to be a chief threat aspect for AD as it increases the enduring risk by almost 2-fold [15]. Early accumulation of Aβ is showed to be partially responsible for CNS insulin resistance and impaired insulin signaling which in turn initiates the brain injury *via* inflammatory and oxidative stress processes thus leading to surfacing of both the diseases.

Moreover, oxidative stress is one of the earliest events in the pathogenesis of both AD and T2D [16]. With aging, increased generation of free radicals and a plodding decline in cellular antioxidant defense mechanisms aggravate the oxidative stress. Therefore, the antioxidants that scavenge free radicals have proven to be a treatment option for AD and T2D [17].

The modern medicines used to relieve AD and T2D symptoms have severe side effects and drug resistance after prolonged treatment [18]. Therefore, to avoid this, WHO paid greater attention on developing herbal medicines with improved and safer therapeutic profile. Ayurveda, an ancient Indian medicinal system practiced from 2000 BC in which plants with antidiabetic and CNS protective effects were well documented [19]. However, medicinal plants and their products that extensively prescribed for the treatment of AD and T2D worldwide have no known scientific base of their activity. Hence, medicinal plants have to be evaluated methodically to test their potential to treat both chronic diseases. Thus with a specific objective, a large number of plants were screened for their inhibitory potential on key enzymes in T2D and AD and three plants *Buchanania axillaris* Desr. (Anacardiaceae), *Hemidesmus indicus* Linn. (Asclepiadaceae) and *Rhus mysorensis* Heyne (Anacardiaceae) were identified as most active. Extensive traditional uses and reported pharmacological activities of titled plants are summarized in Table 1. Thus, the present investigation is aimed to assess the biological potentials including anticholinesterase, antidiabetic, antioxidant and neuroprotective activity, kinetics of enzyme inhibition and the phytochemical profiles of methanol extracts and its derived fractions of *B. axillaris* (BA), *H.indicus* (HI) and *R.mysorensis* (RM) to develop potent agents for dual therapy of both AD and T2D.

**Table 1:** Indian medicinal plants used in the present study and their ethnomedicinal uses

Plant name	Local name	Voucher number	Traditional uses	Reported activities
*B.axillaris*	Sara pappu	YVU 15 AGD	Leaves useful in hyperdipsia, burning sensation, cough, braonchitis, dyspepsia, leprosy and constipation [32] Leaf juice is used as expectorant, aphrodisiac, purgative, depurative, blood purifier, thirst-quencher and cures digestive disorders [33] Gum is antidiarhoeal used for treating rheumatism, diarrhea and intercostals [34] Kernels as a brain tonic Aerial parts to cure itch and to remove blemishes [35]	anti-inflammatory [36], cardioprotective [37] and antioxidant activities [38].
*H.indicus*	Suganda pala	YVU 45 AGD	Root used against asthma, loss of appetite, bleeding piles, bronchitis, diabetes, diarrhea, epileptic fits in children, eye diseases, leucorrhoea, leprosy, leucoderma, indigestion, jaundice, chronic rheumatism, respiratory disorders, skin diseases, syphilis, and urinary diseases [39, 40] Plant used as antipyretic, antidiarrhoeal, anti-rheumatic, antidote in snakebite aphrodisiac, blood purifier, cardio tonic, demulcent, diaphoretic, diuretic, and immunosuppressant, purgative and refrigerant [41–43] To improve quality and quantity of sperms [44].	as antimicrobial, antioxidant, antiinflammatory, antipyretic, antioxidant, wound healing, antithrombotic, antiulcerogenic, hypoglycemic, hypolipidemic, renoprotective, hepatoprotective and antidiabetic activities [45]
*R.mysorensis*	Sitha Sundari	YVU 79 AGD	Fruits to treat dysentery Leaves decoction as remedy for itching Whole plant used in the treatment of diabetes [46]	hepatoprotective and antimicrobial activities [47–49].

## Methods

### Plant collection and sample preparation

Titled plants *B.axillaris* (BA), *H.indicus* (HI) and *R.mysorensis* (RM) are widely distributed throughout tropical Asian countries. The aerial parts of plants were collected in October 2013 from Nallamala hills of Kadapa and its surroundings, a place situated in Andhra Pradesh, India. Dr. A. Madhusudana Reddy, Assistant Professor, Department of Botany, Yogi Vemana University identified the harvested materials employed for the purpose. The herbarium specimens with voucher no. YVU15 AGD, YVU45 AGD and YVU79 AGD were deposited in the herbarium of Yogi Vemana University. The plant materials were air dried at 25–30° C for two weeks, weighed, powdered and stored in darkness at –20 °C until further analysis.

### Plant extraction and fractionation

Each ground plant material (100 g) was extracted twice with 500 ml of 90% methanol by soaking for two days. The plant extracts obtained were filtered *in vacuo* through Whatman No.1 filter paper and the extraction process was repeated twice. The combined filtrates were concentrated using rotavapour (Heidolph, Germany) at 30 °C. The obtained methanolic extracts were fractionated sequentially by different solvents based on polarity viz. chloroform, n-butanol and water for decreasing of complexity of extract and for choosing active fraction. Yield percentage for each extract was calculated. The percentage yield of methanolic extracts per 100g of dry weight for BA, HI and RM are 20.58, 15.69 and 16.97, respectively. The percent yield of CHCl_3_, n-BuOH and H_2_O fractions are 3.9, 2.56 & 2.98 for BA; 4.98, 5.69 & 6.98 for HI; 11.12, 7.44 & 7.01 for RM, respectively.

### Cholinesterase enzyme inhibitory activity

The AChE and BuChE inhibition assay was performed spectrophotometrically for methanolic extracts and its derived fractions using acetylthiocholine iodide and butyrylthiocholine iodide (Sigma-Aldrich, USA), respectively as substrate following the method of Ellman et al*.,* with minor changes [20]. In a 96–well plate, 10 *μ*L of enzyme from stock solution (AChE, 2U/mL and BuChE, 2U/mL) was taken and 10 μL of plant extract or fraction (15–150 *μ*g/mL) and 100 μL Phosphate buffer were added to them. To this mixture, 50 μL DTNB solution (3.96 mg DTNB and 1.5 mg sodium bicarbonate dissolved in 10 mL of 200 mM phosphate buffer pH 7.7) was added and incubated for 5 minutes at 25°C. Substrates with the volume of 15 *μ*L (10.85 mg acetylthiocholine iodide or butyrylthiocholine iodide in 5mL phosphate buffer) were added and incubated for further 5 minutes at 25°C. The colour developed due to the formation of 5-thio-2-nitrobenzoate anion was measured at 412 nm. Galantamine, at 0.12, 0.23, 0.46, 0.92, 1.84, 3.68 and 7.37 μg/mL, and the reaction mixture excluding plant sample were taken as positive and negative controls, respectively. The percent of inhibition and IC_50_ values were determined.

### Glucosidase enzyme inhibitory activity

The inhibition of *α*– and β–Glu activity by methanolic extracts and its derived fractions was assayed with the modified method of Shibano et al*.* [21]. *p*-Nitrophenyl *α*-D-glucopyranoside and *p*-nitrophenyl *β*-D-glucopyranoside were used as subtrates for *α*– and β–Glu, respectively. The assay solution with 10 *μ*L extract or fraction at variuos concentrations (15, 30, 90 and 150 *μ*g/mL), 100 *μ*L phosphate buffer pH 6.8 and 50 *μ*L of enzyme (*α*–Glu 0.15 unit/mL or *β*–Glu 0.15 unit/mL) was incubated at 37*°*C for 15 min. Substrates with a volume of 50 *μ*L (0.5 mM *p*-nitrophenyl *α*-D-glucopyranoside or *p*-nitrophenyl β-D-glucopyranoside) were added and incubated at 37*°*C for 15 min. To this, added 50 *μ*L of 200 mM Na_2_CO_3_ to terminate the reaction. Enzyme activity was determined spectrophotometrically at 415 nm through measuring the quantity of *p*-nitrophenol released. The percent of inhibition and IC_50_ values were determined.

### Kinetic study on enzyme inhibition

Kinetic characterization of enzyme inhibitions was performed at different concentrations of the substrate using relevant assay method based on enzyme [22]. Assay mixture (250 μL) contains 145 μL of 200 mM phosphate buffer (pH 7.7), 80 μL of DTNB (in case of AChE and BuChE) and 10 μL of enzyme. Inhibitors with various concentrations 15, 30, 90 and 150 μg/mL were added and incubated for 5 min at 25° C. Then, added 15 μL of substrate at different concentrations (10, 25, 50, 100 μM). Assay without inhibitor was conducted as control.

The double reciprocal plots of 1/V versus 1/[S] were constructed. In order to get inhibition constants (Ki_1_ and Ki_2_), secondary plots were generated with slopes and intercepts versus inhibitor concentrations.

### Antioxidant activity assay

#### ABTS free radical scavenging assay

The 2,2-azinobis[3-ethylbenzthiazoline]-6-sulfonic acid (ABTS) radical scavenging activity of chloroform fraction of methanolic extract of titled plants was evaluated following a procedure developed by Re et al*.,* with minor modifications [23]. Mixture of 2 mM ABTS and 2.45 mM potassium persulfate solutions was stored overnight in the dark at room temperature to produce free radicals. Finally, mixture of 1mL of ABTS radical solution and 3mL of pyrogallol solution in ethanol in the concentration range of 10–30 mg/mL was incubated for 30 min in the dark. After addition of 10 mL of test fraction, change of absorbance was recorded at 734 nm. Trolox was used as a positive control and results were expressed in Trolax equivalents.

#### DPPH radical scavenging assay

Antioxidant activity of active chloroform fraction of methanolic extract of titled plants was estimated through DPPH radical scavenging capability according to the modified method of Sarikurkcu et al*.* [24]. Chloroform fraction (1.5 mL) of each plant at concentrations of 50, 200, and 400 *μ*g/mL was added to 9 mL of the DPPH solution (60 mM). The reaction mixtures were prepared under dim light. After vigorous shaking, the mixtures were incubated in the dark for 30 min. The decrease in the purple color was measured at 517 nm using 96-well microplate reader. Ascorbic acid and methanol were used as positive and negative controls, respectively. The DPPH radical scavenging capacity was expressed as Ascorbic acid equivalents.

#### Cell culture and treatment

Human neuroblastoma SK N SH cells (National Centre for Cell Sciences, Pune, India) were cultured in minimum essential medium (MEM) supplemented with 1 mM non-essential amino acids, 0.5 mM L-glutamine, 0.1 mM sodium pyruvate and 10% FBS and maintained at 37 °C in a humidified atmosphere of 5% CO_2_. When SK N SH cells reached 80% confluence, and then were used in the following in vitro experiments.

#### Cell viability and MTT assay

Cell viability of SK N SH cells was measured by MTT assay as described previously by Jeelan et al*.* [22]. Cultures of SK N SH cells (0.2 × 10^6^ cells per well) were seeded into 96-wellplate containing in 200 mL of medium supplemented with 10% FBS. Fractions at various concentrations in DMSO were added to wells and plate was placed within a humidified CO_2_ incubator with 5% CO_2_ at 37 °C for 24 h. 20 μL of MTT reagent at a final concentration of 5 mg/mL were added each well and incubated for an additional 4 h period in humidified atmosphere. The medium was removed, then insoluble formazan crystals were dissolved in 200 mL of 0.1 N acidic isopropyl alcohol. Calorimetric measurement of MTT reduction was recorded at 570 nm. The optical density of control cells treated with MEM was taken as 100% viability.

#### Neuroprotection and MTT assay

Neuroprotection by chloroform fractions of titled plants was assayed by measuring induced neuronal cell death in SK N SH cells as described earlier [22]. Control and treated SK N SH cells (15-150 μg of fractions) were incubated with MTT for 3 h in a humidified CO_2_ incubator with 5% CO_2_. To induce oxidative stress, added 1.0 mM H_2_O_2_ and incubated within humidified atmosphere for 24 h period. To investigate the increase in cell viability, soluble orange formazan dye formed was quantified spectrophotometrically as performed in the above assay.

### Phytochemical profiling

#### Determination of total phenolic content

Total phenolic content (TPC) in chloroform fractions was quantified by a standard procedure with minor changes using Folin-Ciocalteu (FC) reagent [25]. 35 μL of chloroform fraction (5 mg/ml) and 150 μL of FC reagent (1mg in 10 mL water) were mixed well and waited for 5 min. Then 115 μL of Na_2_CO_3_ (7.5%) solution was added and mixed thoroughly. The resulting solution was allowed to stand for 2 h and absorbance was recorded spectrophotometrically at 765 nm. Gallic acid (0 to 100 *μ*g/mL) was used as standard. TPC was determined as mg gallic acid equivalents (mg GAE/g) with the aid of standard curve resulted for gallic acid.

#### Determination of total flavonoid contents

The amount of flavonoids was estimated according to the method adopted by Karamian and Ghasemlou (2013)[26]. 0.5 mL of chloroform fraction in 95% alcohol (1.5 mL) was mixed thoroughly with 0.1 mL of 10 % aluminium chloride and 0.1 mL of 1 M potassium acetate (CH_3_COOK) then diluted with 2.8 mL of deionized water. The absorbance at 415 nm was read after incubation at room temperature for 40 min. The total flavonoid content was expressed as mg rutin equivalent (mg RE/g) using a standard curve of rutin.

#### Determination of total tannins contents

Total tannins were quantified using a procedure developed by Sun (1998)[20]. 1mL of chloroform fraction and 3mL of acidic methanol was mixed well and allowed to stand for 10 min at room temperature. To this 6 mL of Vanillin HCl reagent was added and measured absorbance at 500 nm. Tannin contents were expressed as mg Catechin equivalents per ml (CE/ml) as Catechin was chosen as the standard.

#### Determination of total terpenoids

Total terpenoid content was determined by adopting a method described by Narayan Ghorai et al*.* [27]. In a tube 160 μL of chloroform fraction was diluted with 1.2 mL of chloroform, thoroughly mixed and allowed to stand for 3 min. To the assay tube, 100 μL conc. H_2_SO_4_ was added slowly and incubated for 2 hrs at room temperature in dark. After decanting the supernatant of reaction mixture, the resultant reddish brown precipitate was dissolved in 1.5 ml of 95% (Vol/Vol) methanol and absorbance was recorded at 538 nm.

#### Determination of total alkaloids

Total alkaloid content was evaluated with a spectrophotometric method using BCG reagent [28]. To prepare BCG solution, 69.8 mg BCG was completely dissolved in 3 ml of 2 N NaOH and 5 ml distilled water by heating, and then diluted to 1000 ml with distilled water. Standard Boldine solution at concentration of 1 mg per ml was made in HCl solution (pH 2.5). Aliquots of boldine solutions, 5 mL of phosphate buffer pH 4.7 and 5 mL of BCG solution were taken into separatory funnels, shaken vigorously and then 5 mL of chloroform was added. The absorbance at 470 nm was noted after yellow complex was formed in chloroform.

#### Statistical analysis

All the tests were performed in triplicate and data were expressed as means ± Standard Error of Mean (S.E.M). Inhibition percent was calculated by comparing the reaction rates for the sample to the negative control. Percentage inhibition values were log-transformed before being subjected to statistical analysis. The IC_50_ values were determined graphically from inhibition curves plotting log inhibitor concentration Vs. percent of inhibition using Microsoft Excel. Statistical comparison were performed by one-way analysis of variance (ANOVA) using SPSS version 10 software (SPSS Inc., Chicago, USA) and the values were considered as statistically significant when p values were less than 0.05 (*p* < 0.05). Kinetic data analysis was performed using Graph Pad Prism software version 5.0 (Graph Pad Software, Inc., San Diego, USA).

## Results

### Extraction Yields (%)

The shade dried, powdered plant material was extracted with 90% methanol in water. The obtained crude methanolic extract was suspended into water and fractionated by successive solvent-solvent extraction with chloroform and n-butanol. Dried yield percent of 90% methanol extract of BA, HI and RM from solvent extraction by soaking was found to be 20.58, 15.69 and 16.97, respectively. The fractionation of methanolic extract produced the chloroform, n-butanol and residual aqueous fractions. Initially, methanol showed the highest extractive capacity. As indicated by data, different extractive capacities of solvents used for fractionation were in descending order of residual aqueous > n-butanol > chloroform fraction of each solvent.

### In Vitro Biological Activity

To the best of our knowledge, present study was the first attempt to evaluate the ability of the methanolic extract and various fractions of BA, HI and RM to act as multifunctional against both AD and T2D. Therefore, a reliable evaluation protocol requires different activity assessments linked to pathophysiological targets to account various mechanisms of anti-AD and T2D action. In the present study, several in vitro assays like cholinesterase inhibitory, *α*– and β–Glu inhibitory, DPPH and ABTS radical scavenging and neuroprotective activities have been used to determine multifunctional ability of titled plants.

### Cholinesterase Inhibitory Activity

**Fig. 1:**
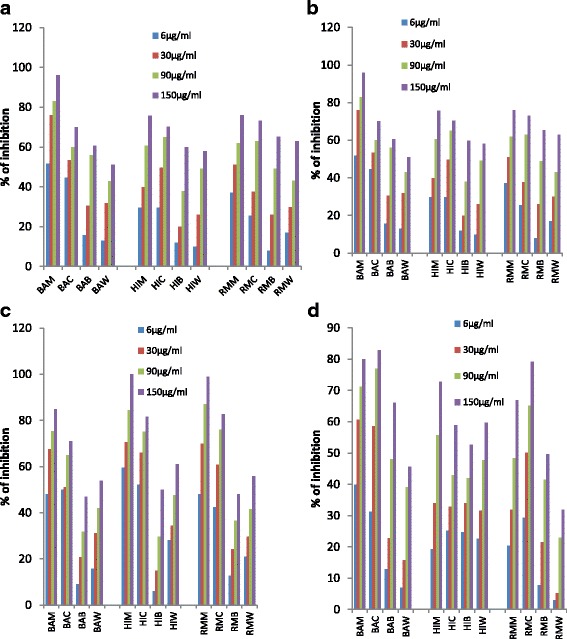
Does dependent inhibitory activity of plant methanolic extracts and its derived fractions against **a** AChE; **b** BuChE; **c** α–Glucosidase; **d** β–Glucosidase

The methanolic extracts of three plants (BAM, HIM and RMM) and its derived fractions (BAC, BAB, and BAW; HIC, HIB, and HIW; RMC, RMB, and RMW) were screened for their inhibitory activity against AChE and BuChE enzymes using Ellman’s colorimetric method [20]. The standard drug Galantamine was used as positive control. All the plant extracts and fractions evaluated at different concentrations (15, 30, 90 and 150 *μ*g/mL) displayed dose-dependent inhibitory activities against enzymes AChE and BuChE (Fig. 1). As tabulated in Table 2, the BAM, HIM and RMM extracts were potent in inhibiting AChE and BuChE with an IC_50_ ranges from 3.8±0.2 to 48.64±11.72 μg/mL (See Additional file 1 for IC_50_ graphs). Among the fractions, BAC, HIC and RMC showed a higher activity than other fractions against AChE and BuChE enzymes with an IC_50_ ranges 5.16±0.22 to 41.35±1.6 μg/mL and were observed as most potent. Thus, all these three plants can be considered as dual inhibitors of AChE and BuChE enzymes. The IC_50_ values of galantamine were 0.77 ± 0.09 and 8.1± 0.02 against AChE and BuChE, respectively. All titled plants showed lesser activity against AChE when compared to galantamine. However, BAM, HIM, HIC and RMM exhibited stronger potency against BuChE than galantamine. Overall, the most prominent AChE inhibition was recorded with BA while HI was most active against BuChE. Comparatively HI and RM showed better activities against BuChE whereas BA was active against AChE. Regarding n-butanol fractions, BAB and RMB showed moderate activity against both the enzymes. In case of water fractions, only HIW displayed moderate activity compared to BAW and RMW against AChE and BuChE.

**Table 2:** IC_50_ Values of 90% methanolic extracts and its derived fractions for AChE, BuChE, α– and β–Glucosidase inhibition assays

Plant	Extract	IC_50_ Values (μg/mL)			
		AChE	BuChE	α–Glc	β–Glc
*B.axillaris*	90% MeOH	4.96 ± 0.96	7.5 ± 0.49	17.14 ± 1.9	25.94 ± 0.4
	CHCl_3_	12.29 ± 2.14	9.94 ± 2.14	16.65 ± 1.99	27.38 ± 1.24
	n-BuOH	67.51 ± 4.81	168.62 ± 39.5	86.61 ± 5.32	270.95 ± 34.09
	H_2_O	136.21 ± 8.2	245.1 ± 35.2	244.66 ± 12.8	387.59 ± 37.8
*H.indicus*	90% MeOH	48.64 ± 11.72	3.8 ± 0.2	9.33 ± 0.2	62.91 ± 6.66
	CHCl_3_	28.14 ± 0.9	5.16 ± 0.22	11.03 ± 0.5	87.64 ± 15.41
	n-BuOH	113.49 ± 11.07	276.74 ± 13.4	220.75 ± 12.4	139.8 ± 15.73
	H_2_O	129.43 ± 8.6	76.62 ± 4.4	70.12 ± 2.13	87.5 ± 5.10
*R.mysorensis*	90% MeOH	21.73 ± 0.3	6.93 ± 0.4	70.11 ± 1.9	32.26 ± 3.4
	CHCl_3_	41.35 ± 1.6	15.86 ± 7.3	26.04 ± 0.37	25.33 ± 0.3
	n-BuOH	83.55 ± 2.8	208.2 ± 22.3	125.61 ± 4.24	238.89 ± 24.88
	H_2_O	93.67 ± 2.3	120.15 ± 14.6	360.07 ± 91.3	590.49 ± 80.9
Galantamine	—	0.77 ± 0.09	8.1 ± 0.02	—	—
Acarbose	—	—	—	117.20 ± 0.017	—
D-Glucono-δ-lactone	—	—	—	—	10.68 ± 0.005

### Inhibition of α– and β–glucosidase enzymes

To assess the antidiabetic potency of titled plants, the extracts and derived fractions were tested for their *α*– and β–Glu inhibitory activity by in vitro enzyme assay as per earlier reported methods [21]. The IC_50_ values of tested extracts and fractions on *α*– and β–Glu are showed in Table 2 (See Additional file 1 for IC_50_ graphs). Acarbose and D-Glucono-δ-lactone were used as reference drugs and showed IC_50_ values ranging between 117.20±0.017 and 10.68±0.005 against *α*– and β–Glu, respectively. The tested methanolic extracts (BAM, HIM and RMM) have shown varying degree of *α*– and β–Glu inhibition with IC_50_ values ranging from 9.33±0.2 μg/mL to 70.11±1.9 μg/mL. Among fractions, the most active was found to be BAC, HIC and RMC with IC_50_ values of 16.65 ± 1.99, 11.03±0.5 and 26.04±0.37 against α–Glu and 27.38±1.24, 87.64±15.41 and 25.33±0.3 on β–Glu. All the titled plants were displayed better potency than the standard acarbose against α–Glu. From these data, it is found that the fractions BAC, HIC and RMC were the most potent in inhibition of *α*– and β–Glu activity. HIW displayed moderate inhibition on *α*– and β–Glu activity; but BAB, BAW, HIB, RMB and RMW fractions were less active against both enzymes

### Kinetic study of inhibition

**Fig. 2:**
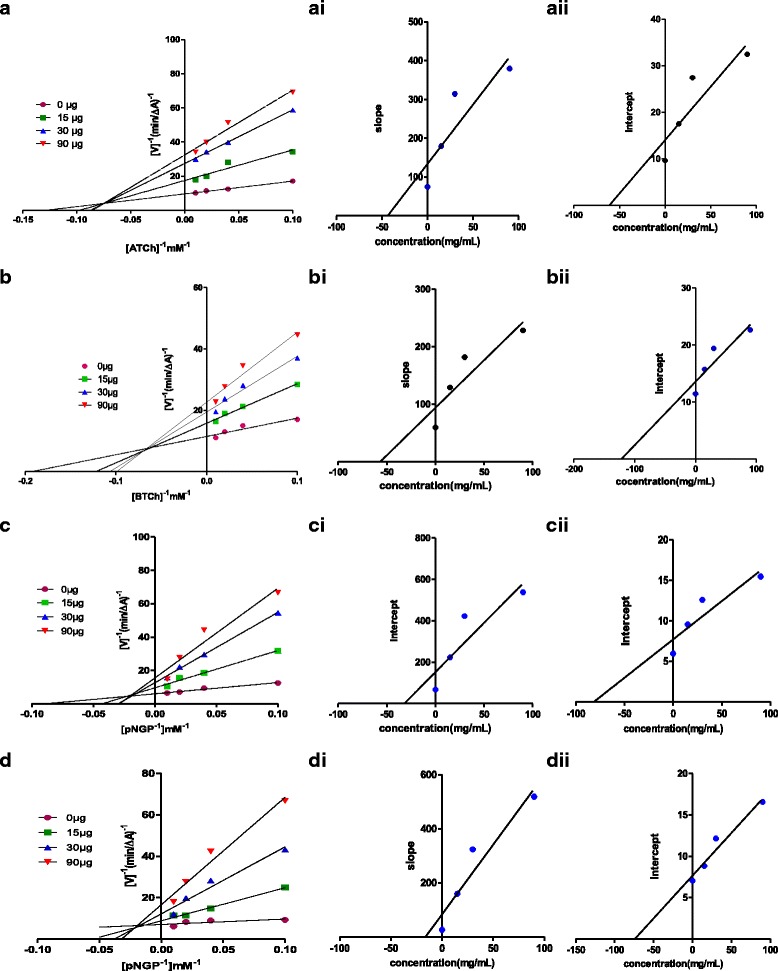
Steady-state inhibition of AChE (**a**), BChE (**b**), α–Glucosidase (**c**) and β–Glucosidase (**d**) by most active fraction (BAC) from *B.axillaris.* (Left) Double reciprocal (1/V vs. 1/S) Lineweaver Burk plots; (right) secondary plots for inhibition constant Ki_1_ (I) (slope vs. various concentrations of BAC) and for inhibition constant Ki_2_ (II) intercept vs. various concentrations of BAC)

In order to explore the mode of inhibition of most active fractions BAC, HIC and RMC, enzyme kinetic studies were conducted. The initial velocity was measured at different concentrations of the substrates (S) using three different concentrations of fractions. The reciprocal of the initial velocity (1/v) was plotted against the reciprocal of concentrations of substrates (Lineweaver-Burke plot) to calculate the rate of the enzyme activity in turn the mode of inhibition (Fig. 2) (See Additional file 1 for kinetic plots of HIC and RMC). At increasing concentration of the inhibitor, increased slopes and intercepts were noticed in Lineweaver-Burke plots. The data points were intersected in the second quadrant (upper left quadrant) of double reciprocal plots. The observations inferred a mixed type of inhibition for most active fractions [29]. For mixed inhibitors BAC, HIC and RMC, two inhibition constants Ki_1_ and Ki_2_ (Table 3) were computed from the secondary plots of slope versus inhibitor concentration and Y-intercept versus inhibitor concentration, respectively. The values of inhibition constants revealed that all active fractions have strong affinity to bind to the free enzyme.

**Table 3:** Kinetic study of most active fractions BAC, HIC and RMC on AChE, BuChE and α- and β-glucosidase enzyme inhibition

S.No	Plant	Extract	Enzymes	Type of inhibition	Inhibition constant (ki) (μg/mL)	
					Ki^1^	Ki^2^
1	*B.axillaris*	CHCl_3_	AChE	Mixed	43.67	61.08
			BuChE	Mixed	56.66	122.3
			α-Glu	Mixed	31.92	81.64
			β-Glu	Mixed	16.03	74.14
2	*H.indicus*	CHCl_3_	AChE	Mixed	19.19	39.68
			BuChE	Mixed	55.69	81.68
			α-Glu	Mixed	66.64	129.01
			β-Glu	Mixed	36.52	51.91
3	*R.mysorensis*	CHCl_3_	AChE	Mixed	71.86	86.31
			BuChE	Mixed	50.11	106.3
			α-Glu	Mixed	38.13	65.92
			β-Glu	Mixed	58.55	99.98

### Antioxidant activity

The antioxidant activity of active fractions was determined using the free radicals DPPH and ABTS by the addition of various concentrations of BAC, HIC and RMC. The total data is tabulated in Table 4.

**Table 4:** Free radical scavenging activity of CHCl_3_fractions using ABTS and DPPH assays

S.No.	Plant	ABTS mg TE /g	DPPH mg AAE / g
1	*B.axillaris*	50.37 ± 1.68	5.97 ± 0.05
2	*H.indicus*	43.9 ± 0.96	5.5 ± 0.2
3	*R.mysorensis*	38.98 ± 4.15	16.4 ± 0.07

Trolox Equivalents, *AAE* Ascorbic acid Equivalents

With respect to ABTS assay, the reduction of ABTS radical cation with hydrogen-donating capacity of fractions was measured spectrophotometrically. All the tested fractions had remarkable radical scavenging activity (RSA) with Trolox Equivalents of 50.37±1.68, 43.9±0.96 and 38.98±4.15 μg/mL.

Concerning DPPH assay, the degree of colour change of purple-coloured DPPH radical solution by electron donation ability of fractions was recorded [30]. In DPPH assay, all the three active fractions BAC, HIC and RMC showed strong RSA with Ascorbic acid Equivalents of 5.97 ± 0.05, 5.5 ± 0.2 and 16.4 ± 0.07 μg/mL, respectively. Ultimately, no marked difference was observed between BAC and HIC in DPPH RSA assay. However, comparatively all the fractions exerted higher activity against ABTS than DPPH indicating that the phytoconstituents of tested fractions have capacity to donate hydrogen to a free radical.

### Cell viability

**Fig. 3:**
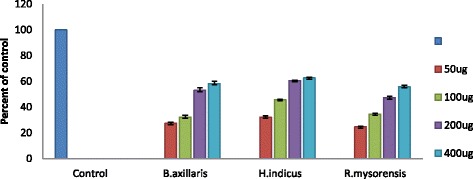
Neurotoxic effects of *B.axillaris, H.indicus* and *R.mysorensis* on SK N SH cells of human neuroblastoma cell line as assessed using MTT assay. Control cells cell viability was taken as 100%

The safety of the extract is absolutely crucial for a successful drug. To grab this, the possible toxicity effects of active fractions BAC, HIC and RMC in the SK N SH cells (human neuroblastoma cell line) was measured using MTT assay. The evaluation results were summarized in Fig. 3. After 24 h incubation, fractions BAC, HIC and RMC at various concentrations (50, 100, 200 and 400 μg) displayed concentration dependent cell viability.

In all, and based on the results obtained, under the above conditions fractions BAC, HIC and RMC showed the cell viability in the range of 25 to 63%. All tested fractions attenuated the cell toxicity significantly in a concentration dependent manner.

### Neuroprotective capacities against H_2_O_2_ induced cell death in SK N SH cells

**Fig. 4:**
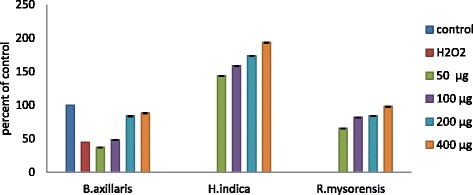
Neuroprotective activity of chloroform fractions from methanolic extracts of *B.axillaris, H.indicus* and *R.mysorensis* on SK N SH cells (human neuroblastoma cell line) against H_2_O_2_ induced cell death in SK N SH cells in MTT assay

The neuroprotective effects of active fractions against H_2_O_2_ induced oxidative stress in SK N SH cells were evaluated using MTT assay. When SK N SH cells were exposed to H_2_O_2_, the cell viability was greatly reduced in concentration and time-dependent way. The survival rate of SK N SH cells was about 44.65 % when the cells were treated with 1.0 mM of H_2_O_2_ for 8 h. On pretreatment with BAC, HIC and RMC at 50, 100, 200 and 400 μg, 24 h before exposure to H_2_O_2_, the viability of SK N SH cells was significantly increased in a dose dependent manner (Fig. 4). When the neuroprotective effect induced by fractions was compared with control, very interestingly, the fraction HIC showed higher neuroprotectivity than control at all concentrations. Fractions BAC and RMC provided almost similar neuroprotective profile to that of control at higher concentrations.

### Phytochemical analyses

Phytochemicals such as polyphenols, flavonoids, alkaloids, terpenoids, tannins etc. that are obtained from plant sources have excellent potential to combat chronic diseases besides promising health promoting properties while acting in combination. Thus, phytochemical analysis reveals significant information about medicinal value of plants.

Phytochemical studies on the most active fractions of titled plants revealed the presence of phenolics, flavonoids, tannins, terpenoids, and alkaloids. Among quantified constituents, the total flavonoid and total phenolic content was significantly higher in all three active fractions BAC, HIC and RMC. Total phenolic contents (TPC) in the active fractions were determined using Folin-Ciocalteu method and expressed as gallic acid equivalents (GAE/g extract). Aluminium-flavonoids complex formation assay was used for the quantification of total flavonoids (TFC). The TFC was expressed as mg RE/100 g dw of extract.

Based on the data expressed in Table 5 the highest amount of flavonoids (691.6±63.26 mg RE/g dry matter) was observed in BAC while the lowest amount (156.32±13.5 mg RE/g dry matter) was detected in HIC. The phenolic content in all the plant species ranged from 182.93±29.3 to 47.12±0.07 mg GAE/g dry matter. The highest amount of phenolics was observed in BAC with 182.93±29.3 GAE/g dry matter. However, almost equal quantities of terpenoids (162.1±1.06 mg LE/g) and flavonoids (156.32±13.5 mg RE/g dry matter) were noticed in HIC. Least amount of tannins and alkaloids were detected in all three fractions. Among tested highest amount of alkaloids (76.51±5.8 mg AE/g) were found in HIC.

**Table 5:** Quantitative phytochemical analysis of the active fractions

Plant	TPC mg GAE / g	TFC mg RE/ g	TTC mg CE/g	TTRC mg LE/g	TAC mg AE/g
*B.axillaris*	182.93 ± 29.3	691.6 ± 63.26	16.936 ± 0.54	17.2 ± 0.52	3.81 ± 0.02
*H.indicus*	47.12 ± 0.07	156.32 ± 13.5	1.576 ± 0.066	162.1 ± 1.06	76.51 ± 5.8
*R.mysorensis*	84.8 ± 2.6	454.93 ± 41.43	9.502 ± 0.22	16.4 ± 0.88	4.35 ± 0.24

*TPC* total phenolic content, *TFC* total flavonoid content, *TTRC* total terpenoid content, *TAC* total alkoloid content, *GAE* Gallic acid equivalents, *RE* Rutin equivalents, *CE* Catechin equivalents, *LE* Linalool equivalents, *AE* Atropin equivalen

### Correlation between total phenolic and flavonoid content and biological activities

**Fig. 5:**
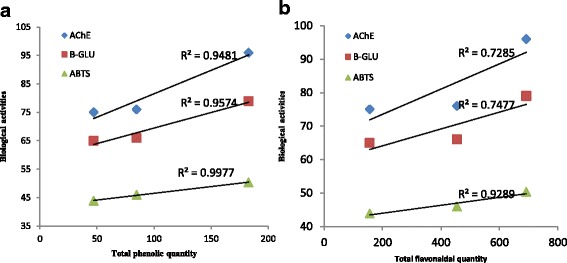
Correlation coefficients (R^2^) of TPC(**a**) and TFC(**b**) in the active fractions of title plants with their biological activities (AChE, β–Glu and ABTS)

The correlation coefficients (R^2^) of the total phenol and biological activities (AChE inhibitory, β–Glu inhibitory and ABTS radical scavenging activities), and total flavonoid and biological activities of the BAC, HIC and RMC were shown in Fig. 5. The R^2^ value of total phenol and AChE inhibitory, β–Glu inhibitory and ABTS radical scavenging activities of BAC, HIC and RMC was 0.94. 0.95 and 0.99, respectively (Fig. 5a). Similarly, R^2^ value of total flavonoid and AChE inhibitory, β–Glu inhibitory and ABTS radical scavenging activities of BAC, HIC and RMC was 0.72, 0.74 and 0.92, respectively. From these data, it is evident that there are high correlations between AChE inhibitory, β–Glu inhibitory and ABTS radical scavenging activities and the total phenol and also the total flavonoid content.

## Discussion

In this study, the crude methanolic extracts of titled plants were fractionated using polar and non-polar solvents to obtain phytoconstituent rich biologically active fractions and provided phytochemical profiling of active fraction. Subsequently, biological evaluations of extracts and fractions against various targets related to AD and T2D were made to prove BA, HI and RM as multifunctional agents for dual therapy for the first time.

Upholding ACh levels by reducing its metabolism in the synaptic cleft by inhibition of ChEs is beneficial for improvement in memory and cognitive dysfunction. Therefore, dual inhibition of AChE and BuChE, a regulatory and a co-regulator enzymes, respectively of cholinergic neurotransmission is continuously deliberated as the “gold standard” therapeutic strategy for the management of AD. Superior dual inhibitory potential of BAC, HIC and RMC on AChE and BuChE in Ellman assay indicating their potential to be as an alternative for the treatment of AD. Inhibition of α- and β-glucosidase enzymes which in turn delay in the digestion of carbohydrates is an effective approach for the management of carbohydrate metabolic disorders like T2D. From the strong α- and β-glucosidase inhibitory activities, it is evident that the fractions BAC, HIC and RMC have excellent antidiabetic potency.

According to current notion, the multifactorial biological pathways involved in AD and T2D seem to share oxidative stress as a unifying factor. Oxidative stress may be either due to excessive production of ROS, loss of antioxidant defenses or both. Consequently, scavenging of ROS has become highly beneficial and worthy strategy for the treatment of AD and T2D. Presently, the exerted higher activity against ABTS and DPPH by the fractions BAC, HIC and RMC indicating that the tested fractions have capacity to prevent the potential damage by ROS. Interestingly, in cell viability assay, the escalating cell proliferation at even high concentrations suggested that fractions are nontoxic to SK N SH cells and likely to promote cell survival or delay the natural death of neurons in culture medium. As fractions provided higher or almost similar neuroprotective profile to that of control at higher concentrations, BAC, HIC and RMC are considered to act as potential oxidative suppressors against H_2_O_2_ induced oxidative stress in SK N SH cells.

In phytochemical profiling, all three active fractions BAC, HIC and RMC found to contain highest TFC and TPC. Also there is strong correlations between AChE inhibitory, β–Glu inhibitory and ABTS radical scavenging activities and the TFC and also the TPC. Naturally occurring flavonoids are reported to have wide range of positive effects on human health through antibacterial, antiviral, anti-inflammatory, anticancer, and anti-allergic properties. Flavonoids are also reported to be highly effective scavengers of various free radicals, prevent or slow the progression of AD by interfering with the generation and polymerization of amyloid-β peptides into neurotoxic oligomeric aggregates, by reducing aggregation of tau proteins and also by inhibiting significantly the ChEs [31]. Phenolic compounds have considered to be enormously responsible for the antioxidant activity of plant materials thereby possessing therapeutic potential for AD and T2D. So compared with literature on other extracts of plants, the present results suggested that phenolics and flavonoids alone or in combination may be the major contributors for the antioxidant and enzyme inhibitory activities. In summary, the pharmacological results suggest the potential of BAC, HIC and RMC to be multifunctional therapeutic remedies for the dual therapy of T2D and AD.

## Conclusions

In the light of our findings, it can be concluded that methanolic extract and its derived chloroform fraction of plants screened herein exhibited high inhibitory activity against AChE, BuChE, α– and β–Glc enzymes. The active chloroform fractions have also showed high antioxidant potential and nueroprotective capacity against H_2_O_2_ induced oxidative stress in neuronal cells. Strong activities can be related with the presence of antioxidant constituents such as phenolics and flavonoids. To the best of our knowledge, we herein divulge the first report on cholinesterase and glucosidase inhibitory, radical scavenging and neuroprotective activity of *B. axillaris*, *H. indicus* and *R. mysorensis*. In conclusion, the present study demonstrates the potential of *B. axillaris*, *H. indicus* and *R. mysorensis* as multifunctional therapeutic remedy for the treatment of AD and T2D. The study warrants further investigations to isolate and characterize the active substances from these plants and to explore their potential in combating degenerative diseases such as AD and T2D.

## Additional file

Additional file 1: Kinetic Study *H.indicus* on AChE, BuChE, α-Glucosidase and β-Glucosidase. Kinetic Study *R.mysorensis* on AChE, BuChE, α-Glucosidase and β-Glucosidase. Inhibitory activity study of *B. axillaris* towards AChE. Inhibitory activity study of *B. axillaris* towards BuChE. Inhibitory activity study of *B. axillaris* towards α- Glucosidase. Inhibitory activity study of *B. axillaris* towards β-Glucosidase. Inhibitory activity study of *H.indicus* towords AChE. Inhibitory activity study of *H.indicus* towords BuChE. Inhibitory activity study of *H.indicus* towords α-Glucosidase. Inhibitory activity study of *H.indicus* towords β-Glucosidase. Inhibitory activity study of *R.mysorensis* towords AChE. Inhibitory activity study of *R.mysorensis* towards BuChE. Inhibitory activity study of *R.mysorensis* towards α-Glucosidase. Inhibitory activity study of *R.mysorensis* towards β-Glucosidase. (DOCX 1045 kb)

## Abbreviations

α–Glu: α–glucosidase
β–Glu: β–glucosidase
ABTS: 2,2'-azino-bis(3-ethylbenzthiazoline-6-sulphonic acid)
ACh: Acetylcholine
AChE: Acetylcholinesterase
AD: Alzheimer’s disease
BuChE: Butyrylcholinesterase
T2D: Type 2 Diabetes Mellitus
DPPH: di(phenyl)-(2,4,6-trinitrophenyl)iminoazanium
DTNB: 5,5’-dithio-bis(2-nitrobenzoic acid)
MTT: 3-(4, 5-dimethylthiozol-2-yl)-2,5-diphenyltetrazoliumbromide
RSA: Radical scavenging activity
