# Supplementary material for: Phytochemical profiling and in vitro screening for anticholinesterase, antioxidant, antiglucosidase and neuroprotective effect of three traditional medicinal plants for Alzheimer’s Disease and Diabetes Mellitus dual therapy
Source: BMC Complement Altern Med. 2018 Mar 2;18:77. doi: 10.1186/s12906-018-2140-x (PMC5834903; doi:10.1186/s12906-018-2140-x)
Supplement: Supplementary file 1 — Kinetic Study H.indicus on AChE, BuChE, α-Glucosidase and β-Glucosidase. Kinetic Study R.mysorensis on AChE, BuChE, α-Glucosidase and β-Glucosidase. Inhibitory activity study of B. axillaris towards AChE. Inhibitory activity study of B. axillaris towards BuChE. Inhibitory activity study of B. axillaris towards α- Glucosidase. Inhibitory activity study of B. axillaris towards β-Glucosidase. Inhibitory activity study of H.indicus towords AChE. Inhibitory activity study of H.indicus towords BuChE. Inhibitory activity study of H.indicus towords α-Glucosidase. Inhibitory activity study of H.indicus towords β-Glucosidase. Inhibitory activity study of R.mysorensis towords AChE. Inhibitory activity study of R.mysorensis towards BuChE. Inhibitory activity study of R.mysorensis towards α-Glucosidase. Inhibitory activity study of R.mysorensis towards β-Glucosidase. (DOCX 1045 kb) [file 12906_2018_2140_MOESM1_ESM.docx]

**Phytochemical Profiling and *In vitro* screening for anticholiesterase, antioxidant, antiglucosidase and neuroprotective effect of three traditional medicinal plants for AD and T2D dual therapy**

Kinetic Study H.indicus on AChE, BuChE, α-Glucosidase, and β-Glucosidase

| A  ** | AI  ** | AII  ** |
| --- | --- | --- |
| B   | BI   | BII   |
| C   | CII   | CII   |
| D   | DI   | DII   |

Fig. 1. Steady-state inhibition of AChE (A), BChE (B), α–Glucosidase (C) and β–Glucosidase (D) by most active fraction (BAC) from H.indicus *.* (Left) Lineweaver Burk plot of reciprocal of initial velocities versus reciprocal of acetylthiocholine iodide concentrations (0.1–0.5 mM) in the absence and presence of BAC at 15 µg, 30 µg, 90 µg and 150 µg; (right) secondary plots of the Lineweaver Burk plot, slope versus various concentrations of BAC (I) regarding inhibition

Kinetic Study R.mysorensis on AChE, BuChE, A-Glucosidase, and b-Glucosidase

| A   | AI   | AII   |
| --- | --- | --- |
| B   | BI   | BII   |
| C   | CI   | CII   |
| D   | DI   | DII   |

Fig. 2. Steady-state inhibition of AChE (A), BChE (B), α–Glucosidase (C) and β–Glucosidase (D) by most active fraction (BAC) from R.mysorensis R.mysorensis *.* (Left) Lineweaver Burk plot of reciprocal of initial velocities versus reciprocal of acetylthiocholine iodide concentrations (0.1–0.5 mM) in the absence and presence of BAC at 15 µg, 30 µg, 90 µg and 150 µg; (right) secondary plots of the Lineweaver Burk plot, slope versus various concentrations of BAC (I) regarding inhibition

Inhibitory activity study of *Buchanania axillaris* towards AChE.

| \|  \| MeOH \| CHCl3 \| butanal \| water \| \| --- \| --- \| --- \| --- \| --- \| \| 6 \| 51.69 \| 44.5 \| 15.69 \| 12.98 \| \| 30 \| 76 \| 53.3 \| 30.58 \| 31.87 \| \| 90 \| 83 \| 60 \| 55.98 \| 42.87 \| \| 150 \| 96 \| 70 \| 60.68 \| 51.07 \| \| IC_50_ \| 4.96 \| 15.87 \| 74.33 \| 149.68 \| |  |
| --- | --- | --- | --- | --- | --- | --- | --- | --- | --- | --- | --- | --- | --- | --- | --- | --- | --- | --- | --- | --- | --- | --- | --- | --- | --- | --- | --- | --- | --- | --- | --- |
| \| Con.c \| MeOH \| CHCl3 \| butanal \| water \| \| --- \| --- \| --- \| --- \| --- \| \| 6 \| 46.98 \| 49.87 \| 15.27 \| 12.67 \| \| 30 \| 75.95 \| 55.67 \| 35.98 \| 33.57 \| \| 90 \| 82.97 \| 57.41 \| 56.98 \| 37.98 \| \| 150 \| 95.67 \| 72.95 \| 66.95 \| 55.98 \| \| IC_50_ \| 6.64 \| 8.46 \| 58.21 \| 137.62 \| |  |
| \| Con.n \| MeOH \| CHCl3 \| butanal \| water \| \| --- \| --- \| --- \| --- \| --- \| \| 6 \| 54.98 \| 45.56 \| 16.65 \| 12.21 \| \| 30 \| 79.98 \| 55.39 \| 33.26 \| 33.36 \| \| 90 \| 82.28 \| 61.64 \| 56.65 \| 42.25 \| \| 150 \| 94.69 \| 70.25 \| 60.69 \| 55.59 \| \| IC_50_ \| 3.3 \| 12.56 \| 70.007 \| 121.35 \| |  |

Inhibitory activity study of *Buchanania axillaris* towards BuChE.

| \|  \| MeOH \| CHCl3 \| butanal \| water \| \| --- \| --- \| --- \| --- \| --- \|  \| 6 \| 47.98 \| 49.82 \| 8.98 \| 15.69 \| \| --- \| --- \| --- \| --- \| --- \| \| 30 \| 67.54 \| 51 \| 20.69 \| 30.98 \| \| 90 \| 75.36 \| 65 \| 31.65 \| 41.98 \| \| 150 \| 84.87 \| 71 \| 46.98 \| 53.98 \| \| IC_50_ \| 6.9 \| 9.9 \| 141.46 \| 325.89 \| |  |
| --- | --- | --- | --- | --- | --- | --- | --- | --- | --- | --- | --- | --- | --- | --- | --- | --- | --- | --- | --- | --- | --- | --- | --- | --- | --- | --- | --- | --- | --- | --- | --- |
| \|  \| MeOH \| CHCl3 \| butanal \| water \| \| --- \| --- \| --- \| --- \| --- \|  \| 6 \| 47.98 \| 48.95 \| 7.94 \| 16.94 \| \| --- \| --- \| --- \| --- \| --- \| \| 30 \| 66.28 \| 51.97 \| 21.64 \| 31.68 \| \| 90 \| 74.64 \| 66.64 \| 33.67 \| 44.91 \| \| 150 \| 83.94 \| 70.64 \| 45.91 \| 55.64 \| \| IC_50_ \| 7.2 \| 10.14 \| 117.96 \| 300.66 \| |  |
| \|  \| MeOH \| CHCl3 \| butanal \| water \| \| --- \| --- \| --- \| --- \| --- \| \| 6 \| 54.98 \| 45.56 \| 16.65 \| 12.21 \| \| 30 \| 79.98 \| 55.39 \| 33.26 \| 33.36 \| \| 90 \| 82.28 \| 61.64 \| 56.65 \| 42.25 \| \| 150 \| 94.69 \| 70.25 \| 60.69 \| 55.59 \| \| IC_50_ \| 3.3 \| 12.56 \| 70.007 \| 121.35 \| |  |

Inhibitory activity study of *Buchanania axillaris* towards α- Glucosidase.

| \|  \| MeOH \| CHCl3 \| butanal \| water \| \| --- \| --- \| --- \| --- \| --- \| \| 6 \| 39.87 \| 31.28 \| 12.87 \| 6.98 \| \| 30 \| 60.69 \| 58.47 \| 22.69 \| 15.74 \| \| 90 \| 71.25 \| 76.98 \| 47.98 \| 39 \| \| 150 \| 79.98 \| 82.87 \| 65.98 \| 45.691 \| \| IC_50_ \| 13.57 \| 18.47 \| 87.27 \| 254.25 \| |  |
| --- | --- | --- | --- | --- | --- | --- | --- | --- | --- | --- | --- | --- | --- | --- | --- | --- | --- | --- | --- | --- | --- | --- | --- | --- | --- | --- | --- | --- | --- | --- | --- |
| \|  \| MeOH \| CHCl3 \| butanal \| water \| \| --- \| --- \| --- \| --- \| --- \| \| 6 \| 34.25 \| 33.68 \| 11.54 \| 5.55 \| \| 30 \| 58.39 \| 54.1 \| 22.61 \| 14.95 \| \| 90 \| 71.87 \| 75.62 \| 45.96 \| 38.64 \| \| 150 \| 80.94 \| 82.61 \| 64.37 \| 44.96 \| \| IC_50_ \| 17.79 \| 18.82 \| 95.49 \| 260.6 \| |  |
| \|  \| MeOH \| CHCl3 \| butanal \| water \| \| --- \| --- \| --- \| --- \| --- \| \| 6 \| 40.58 \| 32.25 \| 11.28 \| 5.54 \| \| 30 \| 61.69 \| 48.94 \| 25.56 \| 16.63 \| \| 90 \| 72.25 \| 79.95 \| 48.94 \| 40.39 \| \| 150 \| 80.12 \| 86.69 \| 68.69 \| 46.69 \| \| IC_50_ \| 20.06 \| 12.67 \| 77.09 \| 219.14 \| |  |

Inhibitory activity study of *Buchanania axillaris* towards β-Glucosidase.

| \|  \| MeOH \| CHCl3 \| butanal \| water \| \| --- \| --- \| --- \| --- \| --- \| \| 6 \| 30.58 \| 33.64 \| 3.64 \| 9.87 \| \| 30 \| 48.79 \| 40.58 \| 13.78 \| 15.84 \| \| 90 \| 65.98 \| 69.87 \| 35.98 \| 31.87 \| \| 150 \| 77.24 \| 84.75 \| 41.69 \| 42.84 \| \| IC_50_ \| 26.62 \| 24.9 \| 335.3 \| 493.74 \| |  |
| --- | --- | --- | --- | --- | --- | --- | --- | --- | --- | --- | --- | --- | --- | --- | --- | --- | --- | --- | --- | --- | --- | --- | --- | --- | --- | --- | --- | --- | --- | --- | --- |
| \|  \| MeOH \| CHCl3 \| butanal \| water \| \| --- \| --- \| --- \| --- \| --- \| \| 6 \| 33.25 \| 30.57 \| 2.24 \| 9.99 \| \| 30 \| 45.67 \| 41.39 \| 11.25 \| 14.69 \| \| 90 \| 66.98 \| 60.97 \| 37.58 \| 33.67 \| \| 150 \| 79.58 \| 88.67 \| 44.95 \| 44.29 \| \| IC_50_ \| 25.23 \| 28.39 \| 262.2 \| 400.75 \| |  |
| \|  \| MeOH \| CHCl3 \| butanal \| water \| \| --- \| --- \| --- \| --- \| --- \| \| 6 \| 31.25 \| 32.17 \| 3.5 \| 10.11 \| \| 30 \| 49.69 \| 41.25 \| 15.64 \| 19.95 \| \| 90 \| 66.34 \| 70.16 \| 38.69 \| 38.64 \| \| 150 \| 75.69 \| 86.94 \| 43.69 \| 49.54 \| \| IC_50_ \| 25.98 \| 28.85 \| 215.35 \| 268.28 \| |  |

Inhibitory activity study of *H.indicus* towords AChE

| \| 6 \| 29.57 \| 29.58 \| 11.87 \| 9.87 \| \| --- \| --- \| --- \| --- \| --- \| \| 30 \| 39.87 \| 49.57 \| 19.87 \| 25.98 \| \| 90 \| 60.69 \| 64.98 \| 37.87 \| 48.98 \| \| 150 \| 75.74 \| 70.25 \| 59.87 \| 57.98 \| \| IC_50_ \| 35.56 \| 29.83 \| 101.59 \| 143.05 \| |  |
| --- | --- | --- | --- | --- | --- | --- | --- | --- | --- | --- | --- | --- | --- | --- | --- | --- | --- | --- | --- | --- | --- | --- | --- | --- | --- | --- |
| \| 6 \| 27.58 \| 25.64 \| 10.47 \| 8.95 \| \| --- \| --- \| --- \| --- \| --- \| \| 30 \| 39.57 \| 52.67 \| 22.67 \| 27.56 \| \| 90 \| 57.69 \| 70.91 \| 34.98 \| 45.67 \| \| 150 \| 76.92 \| 76.95 \| 62.97 \| 59.37 \| \| IC_50_ \| 38.34 \| 26.56 \| 103.26 \| 131.94 \| |  |
| \|  \| MeOH \| CHCl3 \| butanal \| water \| \| --- \| --- \| --- \| --- \| --- \| \| 6 \| 18.64 \| 29.68 \| 29.98 \| 23.64 \| \| 30 \| 34.65 \| 33.64 \| 38.24 \| 35.51 \| \| 90 \| 56.69 \| 44.29 \| 44.45 \| 50.51 \| \| 150 \| 79.98 \| 59.97 \| 55.34 \| 60.58 \| \| IC_50_ \| 75.23 \| 76.83 \| 121.64 \| 77.34 \| |  |

Inhibitory activity study of *H.indicus* towords BuChE

| \| 6 \| 59.54 \| 52.12 \| 5.98 \| 27.98 \| \| --- \| --- \| --- \| --- \| --- \| \| 30 \| 70.45 \| 66 \| 14.75 \| 34.47 \| \| 90 \| 84.45 \| 75 \| 29.58 \| 47.63 \| \| 150 \| 99.98 \| 81.57 \| 49.88 \| 60.98 \| \| IC_50_ \| 3.41 \| 4.91 \| 249.92 \| 85.45 \| |  |
| --- | --- | --- | --- | --- | --- | --- | --- | --- | --- | --- | --- | --- | --- | --- | --- | --- | --- | --- | --- | --- | --- | --- | --- | --- | --- | --- |
| \| 6 \| 56.21 \| 51.69 \| 4.67 \| 25.64 \| \| --- \| --- \| --- \| --- \| --- \| \| 30 \| 72.25 \| 65.95 \| 13.47 \| 33.75 \| \| 90 \| 83.54 \| 75.61 \| 28.94 \| 46.95 \| \| 150 \| 97.39 \| 80.19 \| 50.14 \| 66.64 \| \|  \| 4.04 \| 4.96 \| 291.25 \| 71.04 \|   IC_50_ |  |
| \| 6 \| 27.364 \| 30.24 \| 10.25 \| 10.25 \| \| --- \| --- \| --- \| --- \| --- \| \| 30 \| 40.68 \| 50.68 \| 20.58 \| 22.25 \| \| 90 \| 62.62 \| 65.54 \| 37.89 \| 45.56 \| \| 150 \| 77.54 \| 71.21 \| 60.61 \| 58.94 \| \| IC_50_ \| 72.03 \| 28.03 \| 135.62 \| 113.3 \| |  |

Inhibitory activity study of *H.indicus* towords α-Glucosidase

| \|  \| MeOH \| CHCl3 \| butanal \| water \| \| --- \| --- \| --- \| --- \| --- \| \| 6 \| 47.98 \| 40.98 \| 10.1 \| 21.74 \| \| 30 \| 60.87 \| 65.52 \| 21.12 \| 38.85 \| \| 90 \| 71.15 \| 78.94 \| 34.52 \| 50.14 \| \| 150 \| 87.96 \| 88.36 \| 45.65 \| 61.56 \| \| IC_50_ \| 8.84 \| 10.99 \| 319.56 \| 71.002 \| |  |
| --- | --- | --- | --- | --- | --- | --- | --- | --- | --- | --- | --- | --- | --- | --- | --- | --- | --- | --- | --- | --- | --- | --- | --- | --- | --- | --- | --- | --- | --- | --- | --- |
| \|  \| MeOH \| CHCl3 \| butanal \| water \| \| --- \| --- \| --- \| --- \| --- \| \| 6 \| 46.59 \| 39.65 \| 11.58 \| 20.98 \| \| 30 \| 59.21 \| 63.68 \| 20.87 \| 35.67 \| \| 90 \| 76.58 \| 77.68 \| 31.57 \| 45.24 \| \| 150 \| 85.34 \| 80.97 \| 50.95 \| 66.57 \| \| IC_50_ \| 9.62 \| 12.14 \| 276.63 \| 73.31 \| |  |
| \|  \| MeOH \| CHCl3 \| butanal \| water \| \| --- \| --- \| --- \| --- \| --- \| \| 6 \| 45.98 \| 41.98 \| 11.69 \| 22.67 \| \| 30 \| 61.68 \| 66.94 \| 22.25 \| 38.69 \| \| 90 \| 72.69 \| 79.92 \| 35.68 \| 51.15 \| \| 150 \| 85.69 \| 87.39 \| 46.67 \| 62.94 \| \| IC_50_ \| 9.55 \| 10.09 \| 66.06 \| 293.42 \| |  |

Inhibitory activity study of *H.indicus* towords β-Glucosidase

| \|  \| MeOH \| CHCl3 \| butanal \| water \| \| --- \| --- \| --- \| --- \| --- \| \| 6 \| 19.25 \| 25.14 \| 24.68 \| 22.57 \| \| 30 \| 33.95 \| 32.84 \| 33.87 \| 31.49 \| \| 90 \| 55.69 \| 42.86 \| 41.87 \| 47.65 \| \| 150 \| 72.85 \| 58.94 \| 52.69 \| 59.67 \| \| IC_50_ \| 52.32 \| 118.06 \| 171.2 \| 93.53 \| |  |
| --- | --- | --- | --- | --- | --- | --- | --- | --- | --- | --- | --- | --- | --- | --- | --- | --- | --- | --- | --- | --- | --- | --- | --- | --- | --- | --- | --- | --- | --- | --- | --- |
| \|  \| MeOH \| CHCl3 \| butanal \| water \| \| --- \| --- \| --- \| --- \| --- \| \| 6 \| 15.69 \| 12.39 \| 22.64 \| 22.58 \| \| 30 \| 31.49 \| 31.57 \| 30.97 \| 30.67 \| \| 90 \| 50.97 \| 55.69 \| 44.59 \| 48.97 \| \| 150 \| 72.85 \| 64.57 \| 55.83 \| 59.69 \| \| IC_50_ \| 61.18 \| 68.04 \| 126.75 \| 91.63 \| |  |
| \|  \| MeOH \| CHCl3 \| butanal \| water \| \| --- \| --- \| --- \| --- \| --- \| \| 6 \| 18.64 \| 29.68 \| 29.98 \| 23.64 \| \| 30 \| 34.65 \| 33.64 \| 38.24 \| 35.51 \| \| 90 \| 56.69 \| 44.29 \| 44.45 \| 50.51 \| \| 150 \| 79.98 \| 59.97 \| 55.34 \| 60.58 \| \| IC_50_ \| 75.23 \| 76.83 \| 121.64 \| 77.34 \| |  |

Inhibitory activity study of *R.mysorensis* towords AChE

| \| 6 \| 37 \| 25.47 \| 7.98 \| 16.98 \| \| --- \| --- \| --- \| --- \| --- \| \| 30 \| 51 \| 37.58 \| 25.98 \| 29.87 \| \| 90 \| 62 \| 62.87 \| 48.97 \| 42.98 \| \| 150 \| 76 \| 73.09 \| 65.28 \| 62.98 \| \| IC_50_ \| 22.27 \| 40.14 \| 83.5 \| 97.87 \| |  |
| --- | --- | --- | --- | --- | --- | --- | --- | --- | --- | --- | --- | --- | --- | --- | --- | --- | --- | --- | --- | --- | --- | --- | --- | --- | --- | --- |
| \| 6 \| 35.28 \| 24.97 \| 6.94 \| 15.64 \| \| --- \| --- \| --- \| --- \| --- \| \| 30 \| 55.68 \| 35.67 \| 22.68 \| 28.97 \| \| 90 \| 64.69 \| 60.94 \| 44.98 \| 40.28 \| \| 150 \| 72.98 \| 71.39 \| 67.98 \| 66.98 \| \| IC_50_ \| 20.99 \| 44.63 \| 88.46 \| 93.39 \| |  |
| \| 6 \| 35.69 \| 24.26 \| 7.65 \| 17.59 \| \| --- \| --- \| --- \| --- \| --- \| \| 30 \| 52.67 \| 37.79 \| 28.89 \| 30.24 \| \| 90 \| 62.62 \| 63.69 \| 49.67 \| 44.26 \| \| 150 \| 77.29 \| 74.24 \| 65.65 \| 64.28 \| \| IC_50_ \| 21.94 \| 39.29 \| 78.69 \| 89.77 \| |  |

Inhibitory activity study of *R.mysorensis* towards BuChE

| \| 6 \| 45.69 \| 49.68 \| 11.21 \| 21.12 \| \| --- \| --- \| --- \| --- \| --- \| \| 30 \| 72.25 \| 66.69 \| 25.64 \| 30.27 \| \| 90 \| 88.69 \| 76.64 \| 37.19 \| 44.56 \| \| 150 \| 94.36 \| 81.27 \| 50.27 \| 56.67 \| \| IC_50_ \| 7.5 \| 5.9 \| 206.76 \| 121.05 \| |  |
| --- | --- | --- | --- | --- | --- | --- | --- | --- | --- | --- | --- | --- | --- | --- | --- | --- | --- | --- | --- | --- | --- | --- | --- | --- | --- | --- |
| \| 6 \| 47.34 \| 5.64 \| 9.64 \| 21.04 \| \| --- \| --- \| --- \| --- \| --- \| \| 30 \| 77.64 \| 57.98 \| 19.64 \| 30.54 \| \| 90 \| 82.54 \| 72.64 \| 42.15 \| 45.67 \| \| 150 \| 91.24 \| 92.64 \| 51.64 \| 61.68 \| \| IC_50_ \| 5.94 \| 30.18 \| 170.29 \| 94.42 \| |  |
| \| 6 \| 45.69 \| 49.68 \| 11.21 \| 21.12 \| \| --- \| --- \| --- \| --- \| --- \| \| 30 \| 72.25 \| 66.69 \| 25.64 \| 30.27 \| \| 90 \| 88.69 \| 76.64 \| 37.19 \| 44.56 \| \| 150 \| 94.36 \| 81.27 \| 50.27 \| 56.67 \| \| IC_50_ \| 7.5 \| 5.9 \| 206.76 \| 121.05 \| |  |

Inhibitory activity study of *R.mysorensis* towards α-Glucosidase

| \|  \| MeOH \| CHCl3 \| butanal \| water \| \| --- \| --- \| --- \| --- \| --- \| \| 6 \| 20.31 \| 29.34 \| 7.69 \| 2.98 \| \| 30 \| 31.85 \| 50.11 \| 21.47 \| 5.17 \| \| 90 \| 48.36 \| 65.14 \| 41.45 \| 22.98 \| \| 150 \| 66.87 \| 79.21 \| 49.67 \| 31.86 \| \| IC_50_ \| 72.58 \| 26.62 \| 117.4 \| 337.58 \| |  |
| --- | --- | --- | --- | --- | --- | --- | --- | --- | --- | --- | --- | --- | --- | --- | --- | --- | --- | --- | --- | --- | --- | --- | --- | --- | --- | --- | --- | --- | --- | --- | --- |
| \|  \| MeOH \| CHCl3 \| butanal \| water \| \| --- \| --- \| --- \| --- \| --- \| \| 6 \| 21.65 \| 25.35 \| 5.27 \| 1.12 \| \| 30 \| 30.58 \| 52.67 \| 22.25 \| 5.69 \| \| 90 \| 50.64 \| 64.98 \| 33.68 \| 20.4 \| \| 150 \| 68.62 \| 85.69 \| 50.64 \| 36.98 \| \| IC_50_ \| 66.18 \| 26.15 \| 106.08 \| 214.35 \| |  |
| \| 6 \| 45.69 \| 49.68 \| 11.21 \| 21.12 \| \| --- \| --- \| --- \| --- \| --- \| \| 30 \| 72.25 \| 66.69 \| 25.64 \| 30.27 \| \| 90 \| 88.69 \| 76.64 \| 37.19 \| 44.56 \| \| 150 \| 94.36 \| 81.27 \| 50.27 \| 56.67 \| \| IC_50_ \| 7.5 \| 5.9 \| 206.76 \| 121.05 \| |  |

Inhibitory activity study of *R.mysorensis* towards β-Glucosidase

| \|  \| MeOH \| CHCl3 \| butanal \| water \| \| --- \| --- \| --- \| --- \| --- \| \| 6 \| 31.11 \| 37.17 \| 2.17 \| 4.87 \| \| 30 \| 42.98 \| 44.69 \| 7.94 \| 19.387 \| \| 90 \| 59.87 \| 64.98 \| 16.84 \| 29.87 \| \| 150 \| 67.51 \| 72.98 \| 35.87 \| 36.98 \| \| IC_50_ \| 39.001 \| 25.87 \| 276.04 \| 671.4 \| |  |
| --- | --- | --- | --- | --- | --- | --- | --- | --- | --- | --- | --- | --- | --- | --- | --- | --- | --- | --- | --- | --- | --- | --- | --- | --- | --- | --- | --- | --- | --- | --- | --- |
| \|  \| MeOH \| CHCl3 \| butanal \| water \| \| --- \| --- \| --- \| --- \| --- \| \| 6 \| 30.58 \| 35.24 \| 2.17 \| 4.58 \| \| 30 \| 48.95 \| 45.55 \| 7.94 \| 20.14 \| \| 90 \| 60.19 \| 66.98 \| 16.84 \| 30.25 \| \| 150 \| 73.95 \| 74.25 \| 35.87 \| 39.97 \| \|  \| 30.32 \| 25.38 \| 249 \| 671.4 \|   IC_50_ |  |
| \|  \| MeOH \| CHCl3 \| butanal \| water \| \| --- \| --- \| --- \| --- \| --- \| \| 6 \| 33.65 \| 35.64 \| 2.11 \| 5.54 \| \| 30 \| 49.98 \| 45.92 \| 7.89 \| 20.14 \| \| 90 \| 60.64 \| 65.24 \| 19.97 \| 30.06 \| \| 150 \| 72.35 \| 77.19 \| 40.94 \| 42.21 \| \| IC_50_ \| 27.47 \| 24.76 \| 191.63 \| 428.69 \| |  |
